# A Voltage Mode Memristor Bridge Synaptic Circuit with Memristor Emulators

**DOI:** 10.3390/s120303587

**Published:** 2012-03-14

**Authors:** Maheshwar Pd. Sah, Changju Yang, Hyongsuk Kim, Leon Chua

**Affiliations:** 1 Division of Electronics and Information Engineering, Chonbuk National University, Jeonju 561-756, Korea; E-Mails: maheshwarsah@hotmail.com (M.P.S.); ychangju@jbnu.ac.kr (C.Y.); 2 Department of Electrical Engineering and Computer Sciences, University of California, Berkeley, CA 94720, USA; E-Mail: chua@eecs.berkeley.edu

**Keywords:** memristor bridge, non-volatile programming weight, neuron, synapse, synaptic multiplication

## Abstract

A memristor bridge neural circuit which is able to perform signed synaptic weighting was proposed in our previous study, where the synaptic operation was verified via software simulation of the mathematical model of the HP memristor. This study is an extension of the previous work advancing toward the circuit implementation where the architecture of the memristor bridge synapse is built with memristor emulator circuits. In addition, a simple neural network which performs both synaptic weighting and summation is built by combining memristor emulators-based synapses and differential amplifier circuits. The feasibility of the memristor bridge neural circuit is verified *via* SPICE simulations.

## 1. Introduction

Synaptic multiplications between input signals and weights are key operations in neural networks, programmable analog vector matrix multiplication and cellular neural networks. Most of the previous synaptic multiplications are based on the software models [1–4]. While the flexibility of the software-based model is excellent, its processing speed represents a serious bottleneck. The digital accelerating board on which the software version of neural network is a practical option representing a compromise between limited flexibility and a high speed processing [5,6]. However, this approach may not be the solution for the problem of bigger size of neural networks.

There have been some research efforts to build artificial synapses (weights) in neural network chip and analog programmable vector matrix multiplication using CMOS technologies [7–11]. To implement the immense amount of neural processing on a chip, extremely high density of integration technology is needed. This is a very challenging goal and not many successful cases of neural implementations have been reported so far. The cellular neural network [12–16] is one of the successful implementations of analog multiplication circuits.

Most of the synaptic weights implemented with the conventional technologies are volatile. Also, synaptic multiplication between input signal and weight is non-linear. Therefore, introducing a new weighting technology which is nonvolatile and linear is very important for the further development of neuromorphic engineering.

In 2008, HP announced a successful fabrication of a very compact and non-volatile nano scale memory called the memristor [17]. It was originally postulated by Chua [18,19] as the fourth basic circuit elements in electrical circuits. It is based on the nonlinear characteristics of charge and flux. By supplying a voltage or current to the memristor, its resistance can be altered. In this way, the memristor remembers information.

Many of recent researches showed the great potential of memristors in the application of memory, and artificial synapses [20–24]. Cantley *et al.* presented an application of memristor synapse for the Hebbian learning in spiking neural network [21]. Snider demonstrated a memristor-based self organized network employing dedicated connections for inhibitory (negative) weighting [22]. For such application in neural network or cellular neural network, every connection has to be weighted either positively or negatively.

In [24], we demonstrated the architecture of the memristor bridge circuit which is able to perform signed synaptic operations. The study was conducted with the mathematical model of the HP memristor, where the operation of the memristor bridge circuit was verified via software simulation. This study is an extension of the previous research advancing toward the circuit implementation where the architecture of the memristor bridge neuron is built with our memristor emulator circuits [25]. Also, a simple neural network which performs both synaptic weighting and summation is built by combining memristor emulators-based synapses and differential amplifier circuits.

In this paper, the HP TiO_2_ memristor model is introduced in Section 2. In Section 3, a memristor emulator circuit is proposed. Memristor bridge synapses built with memristor emulator circuits are described in Section 4. Simulation results are presented in Section 5. In Section 6 we present our conclusions.

## 2. HP Memristor Models

**Figure 1.**
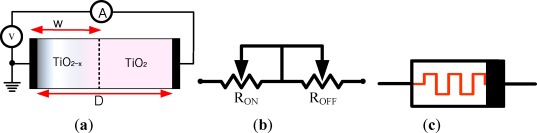
(**a**) Structure of TiO_2_ memristor, TiO_2−x_ and TiO_2_ layers are sandwiched between two platinum electrodes. When a voltage/current is applied, its memristance (resistance of the memristor) is altered; (**b**) equivalent circuit and (**c**) symbol of the memristor.

In HP TiO_2_ memristor model [17], an undoped region with highly resistive TiO_2_ and doped region with highly conductive oxygen vacancies TiO_2−x_ layer are sandwiched between two platinum electrodes as shown in Figure 1(a). When a voltage or current signal is applied to the device, the border line between the doped and undoped layers shifts as a function of the applied voltage or current. In consequence, the resistance between the two electrodes is altered. Figure 1(b,c) is the equivalent circuit and the symbol whose polarity is indicated by a black bar at one end. The defined polarity indicates that the memristance is decreased (or increased) when current flows from the left (right) side to the right (left) side of the memristor symbol in Figure 1(c).

Let *w* be the thickness of the doped area, *D* be the thickness of the two layers of TiO_2_ memristor. Let *R_ON_* and *R_OFF_* denote the minimum resistance and the maximum resistance values, respectively.

Then, the relation between the voltage and the current is given by:

(1) $$v(t)=\left(R_{\mathrm{ON}}\;\frac{w(t)}{D}+R_{\mathrm{OFF}}\;\left(1-\frac{w(t)}{D}\right)\right)i(t)$$

where memristance $M(t)=R_{\mathrm{ON}}\;\frac{w(t)}{D}+R_{\mathrm{OFF}}\;\left(1-\frac{w(t)}{D}\right)$ and *w*(*t*)/*D* is defined as the state variable. In the TiO_2_ memristor [17], the rate of change of the state variable is defined as a function of current *i*; namely:

(2) $$\frac{\mathrm{dw}(t)}{\mathrm{dt}}=\mu_{V}\;\frac{R_{\mathrm{ON}}}{D}\;i(t)$$

where *μ_v_* is the dopant mobility. This model is called a linear drift model, since the velocity of the width is linearly proportional to the current. Integrating Equation (2):

(3) $$w(t)=w_{0}+\mu_{V}\;\frac{R_{\mathrm{ON}}}{D}\int_{0}^{t}i(t)\mathrm{dt}=w_{0}+\mu_{V}\;\frac{R_{\mathrm{ON}}}{D}\;q(t).$$

From Equations (1) and (3), the memristance *M*(*t*) can be written as:

(4) $$M(t)=R_{\mathrm{OFF}}\;\left\{\left[1+\;\frac{w_{0}}{D}\left(\frac{R_{\mathrm{ON}}}{R_{\mathrm{OFF}}}\;-\;1\right)\right]\;-\;\frac{\mu_{v}R_{\mathrm{ON}}}{D^{2}}\left(1\;-\;\frac{R_{\mathrm{ON}}}{R_{\mathrm{OFF}}}\right)q(t)\right\}$$

If *w_0_/D<<1 and R_ON_<<R_OFF_* the expression of *M(t)* is simplified as :

(5) $$M(t)\approx R_{\mathrm{OFF}}\left\{1\;-\;\frac{\mu_{v}R_{\mathrm{ON}}}{D^{2}}q(t)\right\}.$$

*M*(*t*) = *R_OFF_* − *Kq*(*t*), where $K=\frac{\mu_{v}R_{\mathrm{ON}}}{D^{2}R_{\mathrm{OFF}}}.$

From Equation (1):

(6) $$v(t)=\left(R_{\mathrm{OFF}}-\mathrm{Kq}(t)\right)i(t).$$

It follows from Equation (6) that the memristance *M*(*t*) decreases when higher voltage is applied to the non-black bar side than that of black bar side in Figure 1(c). Similarly, the memristor is called incrementally biased when a higher voltage is applied at the black bar side than that of non-black bar side in Figure 1(c). With this bias, the current-voltage relationship is given by:

(7) $$v(t)=\left(R_{0}+\mathrm{Kq}(t)\right)i(t)$$

and the memristance *M*(*t*) increases as *M*(*t*) = *R_o_* + *Kq*(*t*)

Detailed descriptions of incremental and decremental memristors using our emulators circuits are provided in Section 3.

## 3. HP Memristor Emulator Circuit

**Figure 2.**
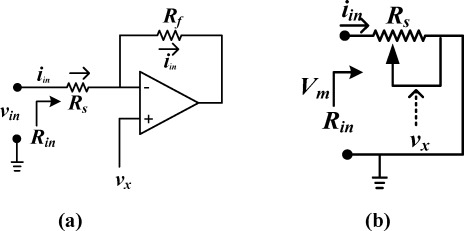
Basic concept for implementing the memristor emulator (**a**) input resistance as a function of voltage *v_x_*; (**b**) equivalent circuit.

As of today, memristors are not yet available on the market. In order to study memristor-based circuit, building memristor emulators is necessary. Two different approaches to build the memristor emulators are the pure analog circuit-based [25] and the analog-digital mixed-based [26,27]. The memristor emulator circuit adopted for this work is from [25]. The basic idea implemented to design the memristor emulator [25] is shown in Figure 2.

In the figure, the voltage at the input terminal is,

(8) $$v_{\mathrm{in}}=R_{s}\;i_{\mathrm{in}}+v_{x}$$

where *i_m_* is the input current, *R_s_* is a resistance at the inverting input terminal and *v_x_* is the voltage applied to the positive terminal of the op Amp.

Assume that the voltage *v_x_* is proportional to input current *i_in_*, then:

(9) $$v_{\mathrm{in}}=R_{s}\;i_{\mathrm{in}}+{\mathrm{mi}}_{\mathrm{in}}=\left(R_{s}+m\right)i_{\mathrm{in}}$$

where *m* is a proportionality coefficient and *v_x_* = *mi_in_*. Equation (9) implies that the input resistance of the circuit is *R_s_* + *m*. If we can control *m* so that, it is time integral of the input current *i_in_*, then, the circuit in Figure 2 acts as a memristor.

To emulate *v_x_* in Equation (9), three devices (a capacitor, a resistor, and a voltage multiplier) are utilized, in which the voltage from the capacitor and that from the resistor are multiplied using a voltage multiplier.

The memristor emulator needs to be prepared in two different connections such as decremental and incremental emulators, separately.

**Figure 3.**
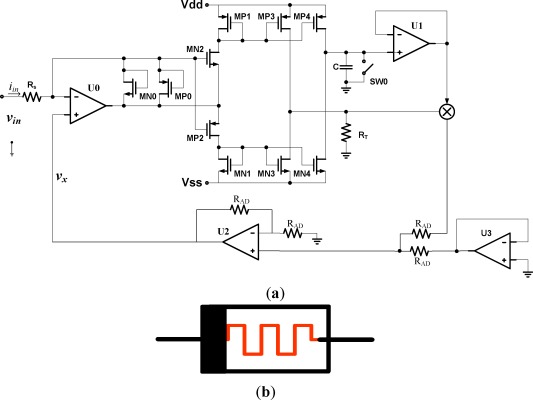
incrementally-biased memristor emulator circuit (**a**) memristor emulator circuit; (**b**) a schematic of memristor emulator.

Figure 3 shows the schematic of the incrementally biased memristor emulator where memristance increases when a positive voltage *v_in_* applied at the input terminal. The input voltage applied at a memristor emulator is converted into an input current *i_in_* with a resistor *R_s_* and op Amp U0 via the virtual ground constraint. Since the current *i_in_* is used at several places, its replicas are generated using current mirrors. Observe that a current mirror copies single directional current only. For bi-directional (positive and negative) currents, *i_in_* must be separated into a positive part and a negative part and processed separately at different parts of the circuit. In the circuit of Figure 3, the positive part of the current, duplicated by a current mirror MN0 and MN2 is fed into a resistor R_T_ and a capacitor C by current mirror MP3 and MP4 with couple of MP1 respectively. On the other hand, MP0 and MP2 acts as the negative part of current mirror that flows out from resistor R_T_ and capacitor C by current mirror MN3 and MN4 which are coupled with MN1.

One of the distinguished features of a memristor is the capability of keeping the programmed information for a long time until new programming inputs are presented. The charge stored at capacitor C is for the programmed information in the memristor emulator. To avoid discharging during the period when an input signal does not exist, the path to the output terminal is connected to a Mosfet buffer U1. The switch S_W0_ is initially closed to reset the capacitor voltage to zero. When a voltage pulse is applied through the input terminal of the emulator circuit, the switch is opened. Therefore the capacitor voltage starts to charge from zero voltage to certain level.

In Figure 3, the capacitor produces a voltage *v_C_* by integrating the current *i_in_*, and the resistor *R_T_* produces a voltage proportional to the current *i_in_*:

(10) $$v_{C}\;=\frac{1}{C}\int i_{\mathrm{in}}\;\mathrm{dt}=\frac{q_{C}}{C},$$

and:

(11) $$v_{R}\;=R_{T}\times i_{\mathrm{in}}.$$

These two voltages are multiplied by a voltage multiplier. The output voltage *v_x_* of the voltage multiplier is given by:

(12) $$v_{x}\;=\frac{q_{C}}{C}\times R_{T}\;i_{\mathrm{in}}.$$

Therefore, the input voltage *v_in_* is:

(13) $$v_{\mathrm{in}}\;=\left(R_{s}+\frac{q_{C}}{C}\times R_{T}\right)i_{\mathrm{in}},$$

where the memristance *M(t*) is:

(14) $$M(t)\;=\left(R_{s}+\frac{q_{C}}{C}\times R_{T}\right).$$

From Equation (14), when a positive pulse is applied at the input terminal, the resistance increases proportional to the time integral of input current with *R_s_*, we call this configuration the incrementally biased memristor which corresponds to the voltage state where the higher voltage is applied at the black bar side of Figure 1(c).

**Figure 4.**
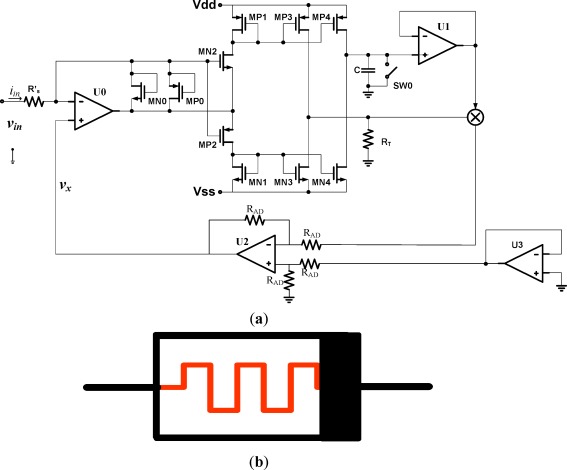
Decrementally-biased memristor emulator circuit (**a**) memristor emulator circuit; (**b**) a schematic of memristor emulator.

On the contrary, if a higher voltage is applied to the non-black bar side, then, the memristance is decreased. We call this configuration the decrementally biased memristor. By adding a voltage inverter after the voltage multiplier as shown in Figure 4, the decrementally biased memristor can be implemented. The input voltage in the decrementally biased memristor is given by:

$$v_{\mathrm{in}}\;=\left(R_{s}^{\prime }-\frac{q_{C}}{C}\times R_{T}\right)i_{\mathrm{in}}.$$

The resultant memristance *M*(*t*) of the decremental memristor is:

(15) $$M(t)=R_{s}^{\prime }-\frac{R_{T}}{C}q(t)=R_{s}^{\prime }\left(1-\frac{R_{T}}{{\mathrm{CR}}_{s}^{\prime }}q(t)\right).$$

## 4. Memristor Neural Circuit Built with Memristor Emulators

**Figure 5.**
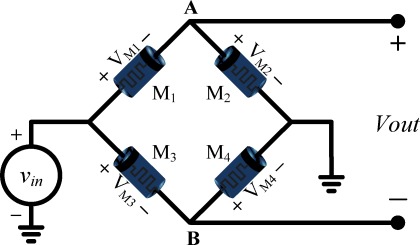
Memristor based synaptic circuit in [24]. It is assumed that M_1_ and M_4_ are decrementally biased memristor while M_2_ and M_3_ are incrementally biased memristors.

The memristor bridge synapse circuit [24] is composed of four memristors as shown in Figure 5. In this study, the architecture of the memristor bridge synapse is built with memristor emulator circuits.

### 4.1. The Memristor Bridge Synapse

When a positive or negative strong pulse *v_in_* is applied at the input terminal of the memristor bridge synapse in Figure 5, the memristance of each memristor is increased or decreased depending upon its polarity.

When a positive pulse is applied at input terminal of Figure 5, the memristances of M_1_ and M_4_ (which are decrementally-biased) decrease. On the other hand, the memristances of M_2_ and M_3_ (which are incrementally-biased) will increase. It follows that the voltage *v*_A_ at node A (with respect to ground) increases while the voltage *v*_B_ at node B decreases. If the pulse width is wide enough, the output voltage *V_out_* varies gradually from negative to positive voltage.

On the other hand, if a negative pulse is applied, when M_1_ and M_4_ are minimum and M_2_ and M_3_ are are their maximum state respectively, then, M_1_ and M_4_ vary to higher memristance and M_2_ and M_3_ go to lower value. It follows that the output voltage *V_out_* varies gradually from positive to negative voltage. In consequence, the weight is able to be programmed with any weights in the range from −1 to +1 including zero using appropriate duration of pulse.

Let *v_in_* be the input voltage pulse. Also, let *V_M1_*, *V_M2_*, *V_M3_*, and *V_M4_* be the voltages across memristor M_1_, M_2_, M_3_, and M_4_ respectively. Then the voltage at each memristor at time *t* is:

(16) $$v_{M1}=\frac{M_{1}}{M_{1}+M_{2}}v_{\mathrm{in}},$$

(17) $$v_{M2}=\frac{M_{2}}{M_{1}+M_{2}}v_{\mathrm{in}}=v_{A},$$

(18) $$v_{M3}=\frac{M_{3}}{M_{3}+M_{4}}v_{\mathrm{in}},$$

(19) $$v_{M4}=\frac{M_{4}}{M_{3}+M_{4}}v_{\mathrm{in}}=v_{B},$$

where M_1_, M_2_, M_3_, and M_4_ denote the corresponding memristance values of the memristors at time *t*, as in Figure 5.

The output voltage *V_out_* of the memristor bridge circuit is equal to the voltage difference between terminal *A* and terminal *B*; namely:

(20) $$V_{\mathrm{out}}=v_{A}-v_{B}=\left(\frac{M_{2}}{M_{1}+M_{2}}-\frac{M_{4}}{M_{3}+M_{4}}\right)v_{\mathrm{in}}$$

where *v*_A_ and *v*_B_ corresponds to the voltages *v_M2_* and *v_M4_*, respectively.

Equation (20) can be rewritten as a relationship:

(21) $$V_{\mathrm{out}}=\xi \times v_{\mathrm{in}},$$

where $\xi =\frac{M_{2}}{M_{1}+M_{2}}-\frac{M_{4}}{M_{3}+M_{4}}$ represents the synaptic weighting factor of the memristor bridge synapse.

### 4.2. Memristor Bridge Synaptic Circuit with Memristor Emulators

The memristor bridge circuit in Figure 5 can be built with memristor emulators which are described in Section 3. In the memristor bridge synapse circuit, the serial connection of two memristors M_1_ and M_2_ are parallel to other serially connected memristors M_3_ and M_4_.

When a voltage pulse is applied at serially connected memristors, the input voltage is distributed to every memristor according to the voltage law so that the sum of each memristor voltage is equal to the input voltage like in ordinary resistors.

**Figure 6.**
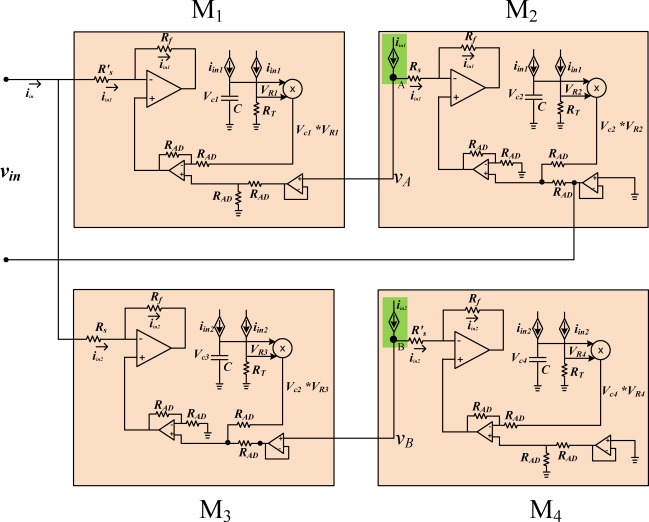
Schematics of memristor emulator-based synaptic circuit corresponding to the synaptic structure of Figure 5.

Figure 6 illustrates the memristor bridge synaptic circuit using four memristor emulators. In this architecture, the input current of the first memristor emulator M_1_ is replicated by a current mirror and fed to the second memristor emulator M_2_ to produce its voltage in the memristor emulator. The voltage produced in the second emulator is added to the first emulator with an analog voltage adder. Therefore, the sum of the individual voltage across each serially connected memristor equals to the input voltage.

### 4.3. Synaptic Multiplication

After the weight setting, the synaptic multiplication between input pulse and weight can be performed by applying a pulse with very narrow width. If the weight is set as in Equation (21), the synaptic multiplication (*V_sm_*) between input pulse(*V_S_*) and weighting factor *(ξ)* is:

(22) $$V_{\mathrm{sm}}=V_{\mathrm{out}}=\xi \times V_{s}.$$

Note that the effect of memristance change is negligible for very narrow pulse signal *V_s_*. Therefore, the weighting factor ξ is constant and output is the linear multiplication between the input pulse and weighting factor ξ. Thus, the memristor bridge circuit acts as a synapse. In case that the memristance change (drift) with weighting operation is really the problem, a doublet circuit can be used to suppress the effect of the memristance change (drift) [28].

**Figure 7.**
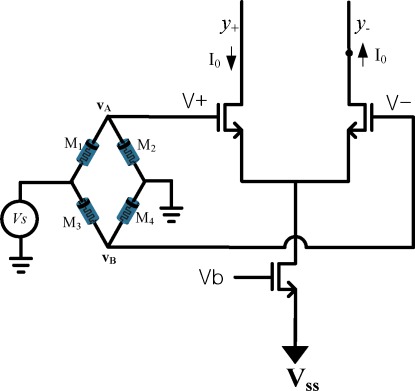
Memristor bridge synaptic circuit. The memristor bridge on the left performs the weighting operation while the differential amplifier on the right performs the voltage to current conversion.

The differential amplifier as shown in Figure 7 is used for voltage to current converter. The output current across differential amplifier for input signal *V_s_* is given as:

(23) $$I_{0}=\frac{g_{m}V_{\mathrm{sm}}}{2}=\frac{g_{m}\xi \times V_{s}}{2}$$

where *g_m_* is the transconductance of Mosfet.

Note that the same input terminal in Figure 7 is shared by the signal *v_in_* for synaptic weight programming and the synaptic input signal *V_s_* for weight processing. The two different kinds of signals are discriminated by being assigned at different time slots.

### 4.4. Memristor Synapse-Based Neural Circuit

The synaptic multiplication in neural network is very important in neuromorphic engineering, programmable analog vector matrix multiplication and CNN circuits [10,11,16].

**Figure 8.**
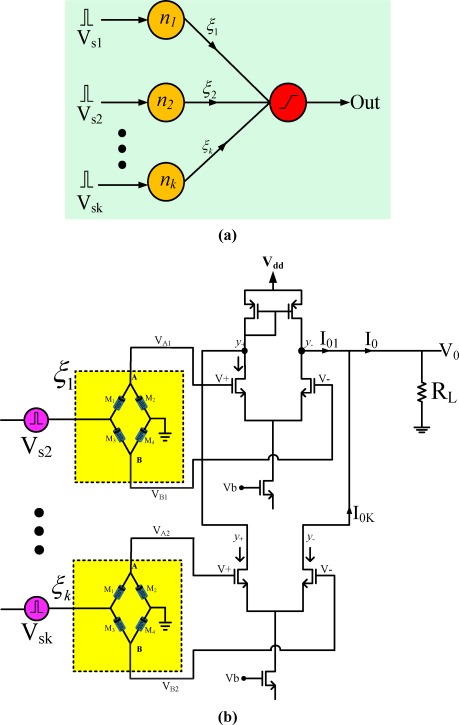
Neural circuit (**a**) Block diagram of single layer neural network (**b**) Memristor synapse-based neural circuit.

Figure 8(a) is a general single layered neural network. The circuit of the memristor synapse-based neuron using memristor bridge and differential amplifier is shown in Figure 8(b). The synaptic multiplications among input pulses and memristor-based weights are conducted in the multiple memristor bridge circuits and the results of the multiplications are summed by simply tying the output terminals in a neuron cell. The sum of the currents is then converted back into a voltage using the load circuit R_L_.

The total current (*I*_0_) at the neuron output is:

$$I_{0}=I_{01}+I_{02}+I_{03}+................I_{0k}.$$

where *I*_0k_, is the output current across differential amplifier corresponding to input voltage pulse *V*_sk_ for *k* th synapse.

The final output voltage across the resistor *R_L_* is given as,

(24) $$V_{0}=I_{0}\times R_{L}=\left[I_{01}+I_{02}+.........I_{0k}\right]R_{L}.$$

From Equations (23) and (24), the output voltage across R_L_ is,

(25) $$V_{0}=\frac{g_{m}R_{L}}{2}\left[\xi_{1}\times V_{s1}+\xi_{2}\times V_{s2}+.........\xi_{n}\times V_{\mathrm{sk}}\right]$$

where *ξ_k_* is the weighting factor of the *k*th synapse.

Therefore, the output voltage of the neuron is given as:

(26) $$V_{0}=\frac{g_{m}R_{L}}{2}\sum_{k=1}^{n}\xi_{k}\times V_{\mathrm{sk}}.$$

Equation (26) reveals that, the output voltage at load resistor *R_L_*, is the weighted sum of the product of each input voltage pulse and programming weight.

## 5. Simulations

In this paper, the memristor bridge architecture [24], is built with memristor emulator circuit. The parameters are chosen as realistic value as possible, so the minimum memristance *R*_ON_ *(R*_S_) *=* 100 Ω, and the maximum memristance *R*_OFF_ *(R'*_s_*) = 16 KΩ*, are taken from those of Stanley Williams’ real memristor [17]. Also, capacitance C and resistance *R*_T_ employed for the memristor emulator are 0.1 μF and *R*_T_ *= 4 KΩ*, respectively. The architecture has been simulated in PSPICE with input voltage pulse ±1 V and power supply ±5 V.

For the weight programming, strong wide pulses were applied to change the state of memristor and very narrow pulses (3 *ns*) were used for synaptic multiplication. The PSPICE simulations were conducted for the weight programming and synaptic multiplication of the memristor emulator-based bridge synapses.

### 5.1. Weight Programming

**Figure 9.**
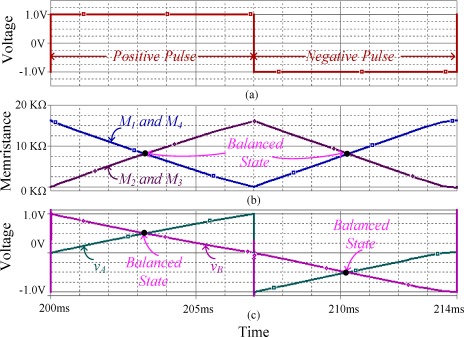
Variation of memristance and voltages (*v_A_, v_B_*) when positive and negative pulses are applied to the emulator-based memristor bridge synapse (**a**) positive and negative input voltage pulses; (**b**) memristance variations; (**c**) voltage variations at *v_A_* and *v_B_*.

Simulations for the weight programming of the memristor emulator-based synaptic circuit as in Figure 6 have been conducted. The synaptic weights were programmed with ±1 V input pulses. Figure 9(b) and Figure 9(c) show the memristance variation and the voltage across each memristor in the memristor bridge circuit for a positive and negative wide pulse.

We assume that the initial memristance of the memristors M_1_ = M_4_ and M_2_ = M_3_ are 16 KΩ(maximum) and 100 Ω(minimum) respectively. Since the polarity of M_1_ and M_4_ are opposite to that of M_2_ and M_3_, the memristances M_1_ and M_4_ decrease, while those of M_2_ and M_3_ increase for positive pulse input, as shown in Figure 9(b). Thus, the voltage *v_A_* increases while *v_B_* decreases as shown in Figure 9(c). When M_1_ = M_2_ = M_3_ = M_4_, *v_A_* equals to *v_B_* and the output voltage becomes zero. At this state, the synaptic weight is zero. When M_1_ or M_4_ is less than M_2_ or M_3_, the voltage *v_A_* is greater than *v_B_*. If the pulse width is sufficiently wide, the voltages at *v_A_* and *v_B_* reach to +1 V and 0 V, respectively. Note that each memristor pair (M_1_, M_4_) or (M_2_, M_3_) is with opposite polarity. Therefore, the composite memristance of each memristor pair is constant.

Similarly, when M_1_, M_4_ and M_2_, M_3_ are in minimum and maximum state respectively, then a negative wide voltage pulse is applied to the memristor bridge synapse, so that the memristance of memristor M_1_, M_4_ and M_2_, M_3_ are moved to the opposite direction compare to the positive case input pulse. In this case, voltage *v_A_* moves toward 0V and that of *v_B_* moves toward −1 V as shown in Figure 9(c).

**Figure 10.**
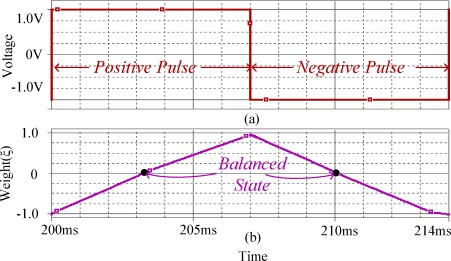
Weight variations of the memristor bridge circuit while positive and negative pulses are applied (**a**) positive and negative input pulses; (**b**) weight variations during each pulse period.

The linearity of the weight programming of the memristor emulator-based memristor bridge synapse has been tested by applying wide positive and negative pulses. The weight values were computed by measuring the output voltages of the memristor bridge circuit while known input voltages were applied, as described in Section 4.1 and 4.2. The results of circuit simulations for the synaptic weighting are shown in Figure 10.

As seen in this simulation result, synaptic weight (ξ) can be changed toward positive (from −1 to +1) and negative direction (+1 to −1) by a positive pulse and negative pulse, respectively. Observe that the programmed weight (ξ) is almost linearly proportional to the width of the input pulse. The linearity of synaptic weight programming in the memristor bridge comes from the complementary action of the back-to-back memristors at each branch of the memristor bridge circuit.

### 5.2. Synaptic Multiplication

**Figure 11.**
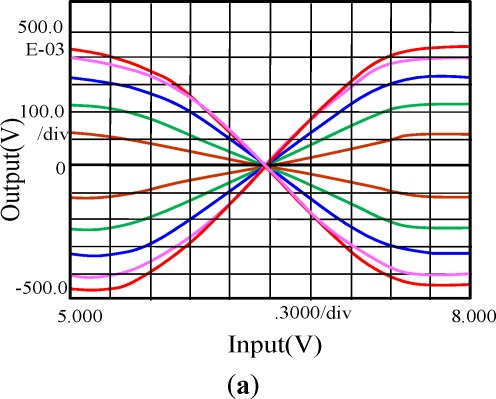

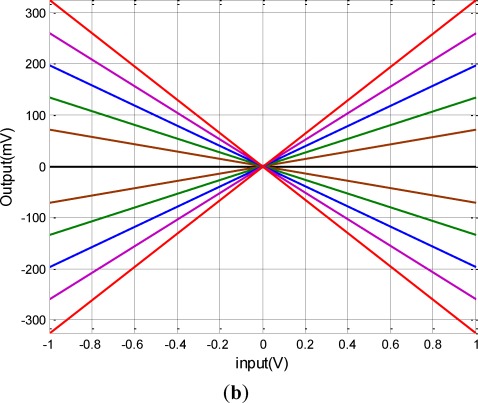
Synaptic multiplication with (**a**) Gilbert multiplier-based circuit [10,16]; (**b**) memristor based circuit.

Simulations of the synaptic weight processing were also conducted with our memrisor emulator-based bridge synapse. Figure 11(b) shows the linearity of the relationship between the input voltages, and the output of the memristor emulator-based bridge synapse. The weighting factor *ξ* is in the range [−0.1,0.1] when synaptic input range is [−1,1] V. The performance of the conventional analog multiplication (synaptic weight) circuit employed in the programmable analog vector matrix multiplication and CNN [10,16] is shown in Figure 11(a). As in the Figure 11(a), the linear region on the function of input-output relation is quite narrow and the intervals between graphs are not quite uniform. However, in the case of memristor bridge synapse, the linear regions are very wide and the intervals between graphs are uniform as in Figure 11(b). The linearity of the memristor bridge synaptic circuit comes from the linear weight assignment at the memristor bridge synapse and the operation at the middle of the memristor dynamic range.

### 5.3. Memristor Synapse-Based Neuron

**Figure 12.**
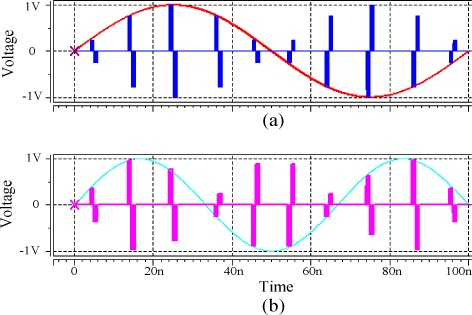

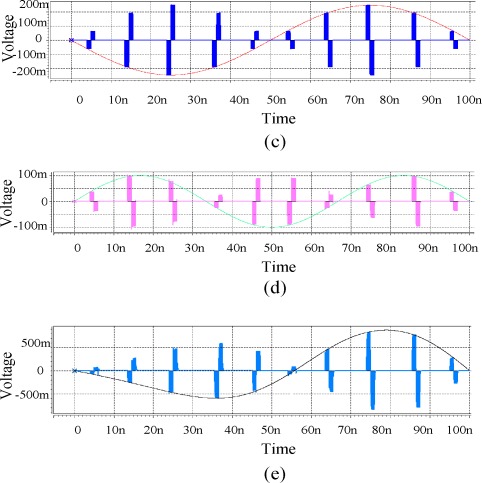
Operations of the memristor emulator-based neuron. Input signals sampled with doublet pulses from two different sinusoidal signals were applied to the memristor bridge synapses, (**a**) input voltage signal for *ξ* = −0.25; (**b**) input voltage signal for *ξ* = 0.1; (**c**) weighted voltage signals with *ξ* = −0.25; (**d**) weighted voltage signals with *ξ* = 0.1 and (**e**) weighted sum appeared at the output of the neuron.

A single layer neuron with two input terminals as in Figure 8(a) has been built with the proposed memristor emulator-based synapse circuit. Two different kinds of sinusoidal voltage signals were sampled by doublet pulses and applied to the memristor synaptic circuits. Figure 12(a–e) are input voltage signals, weighted voltage signals of Figure 12(a,b) with weighting values of *ξ* = −0.25 and 0.1, and weighted sum appeared across *R_L_* where *R_L_* was 10 K.

The use of doublet signals [28] is aimed at preventing the memristances from unwanted drifting. For the subsequent processing with non-memristor circuits, each doublet pulse signal needs to be converted to a singlet pulse. This can be achieved by sampling the output signal at every first pulse period of each doublet. The simulation result shows that the proposed memristor synapse circuit performs synaptic action excellently without significant distortion.

## 6. Conclusions

This paper is the extension of our previous work on memristor bridge synapses [24]. In this paper the mathematical model-based memristor bridge synapse of the previous work is built with memristor emulator-based synapse circuits.

Simulations for the weight programming were performed with memristor emulator-based bridge synapse circuit. The programmed weights were almost linearly proportional to the width of the input pulses. The linearity of weight programming in the memristor bridge synapse comes from the complementary action of the back-to-back memristor pair of the memristor bridge synapse. The simulations of synaptic multiplication between programmed weight and input signal also was conducted. It showed an excellent linearity compared to that of the conventional Gilbert multiplier-based circuit. In the simulation of a single layer neuron, the proposed memristor-based neural circuit performs both synaptic weighting and summing actions very well without significant distortion.

There are several benefits with the proposed memristor synapse circuit over the conventional circuits. The number of transistors required for the memristor based synaptic circuit is three, while that of Gilbert multiplier-based synaptic circuit is seven. Considering the fact that the total size of four memristors with the proposed circuit is less than that of a single transistor, the size benefit of the proposed synaptic circuit is obvious. Also, non-volatility as memory and excellent linearity in synaptic operation are additional benefits of the proposed memristor synaptic circuit.
